# Panel or Anti-Panel? Why Practitioners Must Make Their Choice!

**Published:** 1913-12-13

**Authors:** 


### THE PROFESSION OF MEDICINE.

(Contributions and Correspondence are Invited, that the Grave Questions of the Hour may be Examined from all Points of View.)

### PANEL OR ANTI-PANEL? TRADE OR A LEARNED PROFESSION?

WHY PRACTITIONERS MUST MAKE THEIR CHOICE!

The formation of the National Medical Union, recorded in The Hospital last week, is, of necessity, an organisation for the defence of the profession upon grounds which are largely administrative and financial. At a meeting of promoters the speakers attuned themselves to varied notes in promulgating their needs and securing confidence. Upon the members of the profession will now devolve the duty of declaring their adhesion to principles which separate them from the odium of trade in its less honourable forms, and maintain their standard as members of a learned profession.

### The Heart of the Matter.

The question circles round the provision of needful help for the poor. To no class of men are the needs of the poor so well known as they are to the general practitioner, and from no class of men have they received a more ready and helpful sympathy. Every one of them begins his study for the duties of his life under the guidance of the physicians and surgeons of one or other of our great hospitals, who render to the poor the most skilled service which our land commands, and for a merely nominal monetary return. Their investigations and their results benefit first their hospital patients, and if there is any rivalry it is that their hospital records should be the best that can be obtained. The junior practitioner, imbued with all the information which such teaching can impart, finds his zeal sharpened by sympathy when he meets with familiar, and often unfamiliar, forms of disease hampered and hindered in their course by the conditions daily seen in Poor-Law practice. The club doctor, dealing for a nominal sum with the illness of the breadwinner of the family, formerly gave freely his time and energy to conquer the danger which threatened the future of more than one life.

### The Lessons of Private Practice.

The regard and gratitude which often remained with the patient, when he had outlived his illness and made a success in life, were one of the prizes of the profession. The panel system compels us to speak of these experiences of private practice in the past tense. But they were not the doctor's only reward. The study which saved a life is also a remembrance which is not lost on other cases nor in the formation of his own character. If medicine is to be a learned profession it must be, from first to last, a sympathetic study. No man should select the medical profession as his line in life who is capable of misinterpreting the advice of the late Sir James Simpson to his students, "You must climb over the backs of the poor to reach the pockets of the rich." That is the debt which we owe, and which we ought honourably to pay. We earn much in learning, but it is not money.

From another standpoint, this under-paid service remedied an almost unavoidable fault in hospital education. There is so much to be learnt in the wards during a hospital career that the outdoor dispensary work receives too little attention, and yet it is what is met first in general practice. The late Lord Lister told his students at Glasgow: "You will rush through to Edinburgh to see Mr. Syme tie the iliac artery, which you are never likely to be called upon to do, while few of you, who are not dressers, will attend to see my house surgeon deal with a case of piles." All through this comparatively trivial work there is a reason and a meaning; every detail should be a study, and all treatment the outcome of a careful conclusion. As time goes by this studious attitude becomes a habit; the experience an asset which tends to quickness; and the man an opinion and a personality.

### Tendency of the Panel System.

But the tendency of the panel system is, alas! towards quickness without either study or experience. The prize from the first is to gain a sufficient number of underpaying patients to cover even the working expenses. Clerical details interfere with careful diagnosis and tempt the overworked man to avoid criticism by furnishing unreliable figures for the compilation of worthless statistics. Knowledge cannot advance commensurately with opportunities which must perforce slip by, and the risk of "stop-gap" records, while his conscience lasts, is only too apt to degenerate into an unreasoning quackery which he would have resented in his earlier days. But while the panel lasts what can the panel practitioner do? It robs him of the sympathy and interest which his nature breeds in him towards the deserving poor, by the intrusion of others whom he can only regard as being treated "on the cheap," and to the exclusion of all consciousness of fair dealing.

These detriments are so serious to the good name and honour of Medicine as a learned profession that the National Medical Union should find many supporters. It cannot be denied that means might possibly be found to render some panel system as useful to the development of knowledge and experience as the Poor-Law system or that of the friendly societies has been in the past. This can never be so under the present panel system, and high standards of practice can never be until the work and remuneration are better proportioned. To point out these destructive tendencies of panel practice must surely stiffen the backs of all who desire the maintenance of Medicine as a learned profession. Hence the more honourable and the higher the principles which inspire the practitioner the more is it his duty to at once become a member of the National Medical Union, that he may put forth all his strength in opposition to the degenerating influences of panel practice before they destroy Medicine as a profession and substitute a trade. Meanwhile, who can doubt the view which the public must speedily take of panel practice and some who engage in it?

